# Usability and knowledge testing of educational tools about infant vaccination pain management directed to postnatal nurses

**DOI:** 10.1186/s12909-015-0305-6

**Published:** 2015-03-12

**Authors:** Anna Taddio, Vibhuti Shah, Jane Wang, Chaitya Parikh, Sarah Smart, Moshe Ipp, Rebecca Pillai Riddell, Linda S Franck

**Affiliations:** Clinical Social and Administrative Pharmacy, Leslie Dan Faculty of Pharmacy, University of Toronto, 144 College Street, Toronto, ON M5S 3M2 Canada; Child Health Evaluative Sciences, The Hospital for Sick Children, 555 University Avenue, Toronto, ON M5G 1X8 Canada; Department of Paediatrics, Mount Sinai Hospital, 600 University Avenue, Toronto, ON M5G 1X5 Canada; Undergraduate Pharmacy Division, Leslie Dan Faculty of Pharmacy, University of Toronto, 144 College Street, Toronto, ON M5S 3M2 Canada; Graduate Department of Pharmaceutical Sciences, Leslie Dan Faculty of Pharmacy, University of Toronto, 144, College Street, Toronto, ON M5S 3M2 Canada; Department of Paediatrics, The Hospital for Sick Children, 555 University Avenue, Toronto, ON M5G 1X8 Canada; Department of Psychology, Faculty of Health, York University, 4700 Keele Street, Toronto, ON M3J 1P3 Canada; Department of Family Health Care Nursing, University of California, San Francisco, 2 Koret Way, N411F, Box 0606, San Francisco, CA 94143 USA

**Keywords:** Infant, Vaccination, Pain management, Medical education, Knowledge translation, Postnatal nurse, Parent education, Implementation science

## Abstract

**Background:**

Adapting educational tools to meet user needs is a critical aspect of translating research evidence into best clinical practices. The objectives of this study were to evaluate usability and effectiveness of educational tools about infant vaccination pain management directed to postnatal nurses.

**Methods:**

Mixed methods design. A template pamphlet and video included in a published clinical practice guideline were subjected to heuristic usability evaluation and then the revised tools were reviewed by postnatal hospital nurses in three rounds of interviews involving 8 to 12 nurses per round. Nurses’ knowledge about evidence-based pain management interventions was evaluated at three time points: baseline, after pamphlet review, and after video review.

**Results:**

Of 32 eligible postnatal nurses, 29 agreed to participation and data were available for 28. Three overarching themes were identified in the interviews: 1) utility of information, 2) access to information, and 3) process for infant procedures. Nurses’ knowledge improved significantly (p < 0.05) from the baseline phase to the pamphlet review phase, and again from the pamphlet review phase to the video review phase.

**Conclusions:**

This study demonstrated usability and knowledge uptake from a nurse-directed educational pamphlet and video about managing infant vaccination pain. Future studies are needed to determine the impact of implementing these educational tools in the postnatal hospital setting on parental utilization of analgesic interventions during infant hospitalization and future infant vaccinations.

## Background

Over 90% of young children exhibit serious distress during vaccine injections, defined as a distress score of 3 or greater on a scale of 1 to 5 [1] and both parents and clinicians report being concerned about injection-related distress in children [2-5]. Numerous pain-relieving interventions are available to mitigate vaccination pain [6-8]; however, they are not consistently administered in clinical practice [4,9]. A knowledge-to-care gap therefore exists between what is known about vaccination pain management and what is being done to manage pain during routine vaccinations.

According to the Knowledge-to-Action Framework [10], for scientific evidence to be adopted in clinical practice, best-practice guidelines and educational tools are needed. We developed the first evidence-based clinical practice guideline (CPG) in 2010 to address vaccination pain management in children [11]. Template educational tools were incorporated in the guideline, including a pamphlet and video.

Successful implementation of guideline educational tools requires they be adapted to the local context [10]. This is achieved through an iterative process of obtaining feedback and modifying the tools according to specific needs of the target end-user [10]. In a previous study, we adapted our template tools to suit the needs of new parents and demonstrated significant gains in their knowledge about evidence-based pain management interventions and intentions to use the information at future infant vaccinations [12].

In the present study, we targeted hospital nurses working on the postnatal ward (i.e., postnatal nurses). Postnatal nurses are ideally suited to both reinforce and teach new parents about vaccination pain management interventions. Postnatal nurses routinely educate new parents about infant care and perform painful medical procedures in infants, including immunization injections. In addition, parents have recommended the postnatal setting to learn about vaccination pain management [12]. In the present study, we adapted our clinician-directed tools (pamphlet, video) to postnatal nurses and evaluated usability and knowledge uptake.

## Methods

### Study design

We employed a mixed methods design. The qualitative component consisted of three rounds of individual and group interviews with nurses with a minimum of 8 different nurses per round. The quantitative component consisted of quality assessment and knowledge uptake.

### Participants and setting

A convenience sample of postnatal nurses on the Mother and Baby Unit, Mount Sinai Hospital (MSH) in Toronto and taking care of mother-infant dyads after the delivery of a newborn infant were eligible. All nurses provided newborn care to infants including injections of hepatitis B vaccines and antibiotics. Nurses were trained to use some methods of pain relief during procedures such as breastfeeding; however, there was no unit protocol regarding pain management during painful procedures. There were no specific exclusion criteria. Ethical approval was obtained from the Mount Sinai Hospital Research Ethics Board and all participants signed a written consent form.

### Development and review of educational tools

The template clinician pamphlet published with the CPG comprised of a 2-sided full page sheet describing pain-relieving interventions for use in children of all ages [11]. The front side included coloured pictures; the reverse included coloured pictures with accompanying written instructions. The template video was a 20-minute documentary including an overview of the importance of managing vaccination pain and video vignettes of children of different ages undergoing vaccine injections with and without analgesic interventions (available at: http://www.sickkids.ca/Learning/SpotlightOnLearning/profiles-in-learning/help-eliminate-pain-in-kids/index.html).

Before showing these tools to postnatal nurses, a human factors engineer conducted a heuristic usability evaluation of both materials to determine if design elements followed established principles for user interface design [13]. The heuristic evaluation incorporated broad ‘rules of thumb’ such as; making options and actions easily visible, using words and phrases with clear meaning and familiarity to the user group, and removing irrelevant information. This evaluation led to changes to the pamphlet and video. For the pamphlet, the order of the interventions was altered to coincide with timing prior to vaccine injection (i.e., interventions were ordered according to a timeline leading up to the injection), and some images and text were removed or revised. For the video, the duration was reduced, more specific information about how to implement individual interventions was included, and information was restricted to young children.

The pamphlet was revised after each of the three rounds of interviews conducted with different groups of postnatal nurses according to comments made by the nurses. It was not feasible to make changes to the video during the conduct of the study.

### Study procedures

All interviews with each participant were divided into three phases: baseline, pamphlet review, and video review. During the baseline, nurses were asked about their knowledge and attitudes regarding vaccination pain. Discussion was facilitated by a trained facilitator using a semi-structured interview script. Questions included: 1) What do you know about this topic? 2) How do you want to learn about this topic? 3) What do you do to manage pain in infants undergoing painful medical procedures? 4) How can you help parents to know more about this topic? Then nurses reviewed the pamphlet and the video. The pamphlet phase always preceded the video phase, and in each successive phase, nurses provided feedback about the tool being reviewed using a validated survey instrument that inquired about how much of the information they were able to understand and if the information was adequate [14].

A knowledge test modified from a previous study [12] was administered to each participant during each of the three phases (Table 1). It included 12 true/false questions about the effectiveness of various interventions for reducing vaccination pain; the information for 8 of the questions was featured in the educational material. Nurses rated level of confidence in their responses to each question using a 5-point Likert scale (very sure, a little sure, neither sure/nor unsure, a little unsure, very unsure). The knowledge test included items that were not included in the materials to try to minimize acquiescence bias (i.e., tendency to respond positively or to agree with all the questions when in doubt). We have used this method previously [12].

**Table 1:** **Knowledge test for vaccination pain management in infants**

	Correct response*
1. Giving sugar water can reduce pain and distress.**	True
2. Using medicines like acetaminophen (Tylenol, Tempra), or ibuprofen (Advil, Motrin) can reduce pain and distress.	False
3. Putting ice on the skin can reduce pain and distress.	False
4. Breastfeeding can reduce pain and distress.**	True
5. Bottle feeding can reduce pain and distress.	True
6. Holding the baby can reduce pain and distress.**	True
7. Using numbing (anaesthetic) medicines can reduce pain and distress.**	True
8. Distracting the baby can reduce pain and distress.**	True
9. Acting calm can reduce pain and distress.**	True
10. Rubbing the skin can reduce pain and distress.	False
11. Performing intramuscular injections quickly without prior aspiration can reduce pain and distress.**	True
12. Giving the most painful vaccine first if multiple vaccines are injected sequentially can reduce pain and distress.**	False

*Based on HELPinKIDS clinical practice guideline [11].

**Information featured prominently in the educational material.

### Data analysis

For the qualitative analysis, interviews were audio-recorded and transcribed verbatim and analysed using content analysis [15]. Data collection and analysis occurred simultaneously until saturation of the key emerging themes occurred. Two authors participated in data analysis. The frequency and consistency in which participants indicated categories of responses in the transcripts was used to provide credibility to these categories. The results were reviewed with postnatal nurses to verify interpretation of the data. The qualitative component of the study adheres to the RATS guidelines on qualitative research (http://www.biomedcentral.com/ifora/rats). For the quantitative analyses, the number of correct responses on the knowledge test for each participant at the baseline phase, post-pamphlet phase and post-video phase was analyzed using repeated measures ANOVA. Analyses were repeated including only questions pertaining to the subset of items that were featured in the educational materials. The interview round (1, 2, 3) was included in the analysis as a covariate. The statistical program SPSS version 20 was used. A p-value <0.05 was considered significant.

## Results

The study was conducted between July 1, 2011 and December 22, 2011. Altogether, 32 nurses were approached and 29 (91%) agreed to participate. The mean age was 40 years (standard deviation, SD = 13). All nurses were female. One nurse (3%) did not complete the study procedures due to being called away for clinical duty and is not included in the analysis.

### Qualitative analysis

Overall, three overarching themes were identified from the interviews: 1) utility of information, 2) access to information and 3) process for infant procedures.

1. ***Utility of information***
  Nurses reported that the tools increased their awareness about the importance of managing pain in infants and provided them with the knowledge and skills to be able to carry out better pain management practices.
  *C02: I’m more conscious with my baby when I do my blood work with my baby, like doing anything, any invasive procedure…*
  *C09: A lot of these tools can be used… holding your baby, breastfeeding. So it can be applied in not only vaccinations, but in other areas. Any circle of work, we can use it as teaching opportunities.*
  Nurses reported being motivated to want to more fully involve parents in medical procedures.
  *C09: well in my practice, I’d be more teaching. Preparing (parents) about vaccinations for their newborns. That way they know what to expect and how to react and how to help their babies manage pain… it’s a mini in-service that you gave me. It does make me more empowered, more comfortable with talking about pain management. Because you usually don’t associate newborns and pain management.*
  Nurses compared the information to institutional policies and practices. They were generally receptive to information contained in the tools; however, they identified some inconsistencies with institutional policies and practices and recommended that information be harmonized.
  *C15: …we tend to uh, offer mom to have the baby on the breast, skin to skin and also do finger sucking to help…(and) well for me, um, if its proven that its best practice then I will be very open to put that into my practice. …Not aspirating before intramuscular injection is a new thing for us because it changes our practice. So, I’m really struggling a little bit with that…*
2. ***Access to information***
  All postnatal nurses offered suggestions for peers to access the information, including; e-learning modules; in-services; classroom presentations; research days; staff meetings; hospital television learning channel; on-line hospital policies; mass dissemination of pamphlets, and poster displays.
  *C13: I think maybe making it part of the e-learning…because we all do that yearly. Umm, I think small in-services would be beneficial or at staff meeting because we are all together usually at the same time…*
  Nurses reported strategies for informing parents, including; displaying posters, including the pamphlet in the admissions package, and playing the video in the lounge during discharge classes.
  *C24: …we have an admissions package that we give to [parents] like a folder with a whole bunch of information and it would be great to be able to put this in the admissions package.*
  Some postnatal nurses reported the tools were complimentary while others reported the video was sufficient or superior to the pamphlet:
  *C06: …it is helpful to hear and see rather than just see, or just read, this just combines and is more believable.*
  *C20: I like the video better. I can actually see the actual suggestions working.*
3. ***Process for infant procedures***
  Postnatal nurses described the process for carrying out painful medical procedures in infants. Some reported routinely involving parents and assigning roles such as breastfeeding or pacifying infants with a finger.
  *C16: Well, if I was doing some blood work on the baby, I would …um, explain to the mother that it’s very important that the baby be able to breastfeed and to relax, mother and baby to relax and while the baby does the breastfeeding, I would do the blood work.*
  Others reported taking infants away or asking parents about their preferences about their level of involvement during procedures.
  *C04: …bring the babies here to do the tests, in isolation, in (the) observation area.*
  *C12: I usually (tell) parents, okay, I’m going to (do) blood work…do you want me to take the baby? If you don’t want to see blood… it’s fine, you are not gonna see it…*
  Nurses reported that parents have different preferences regarding being involved in infant procedures.
  *C02: …Its half-half, some parents will say I don’t want to deal with it and some will cuddle the baby.*
  Nurses reported avoiding long explanations and performing procedures quickly in order to minimize parental anxiety.
  C*26: sometimes the patient’s getting more nervous while you’re explaining what kind of procedure you are going to administer now… does not want to see anything, just do it quickly and that’s it…*
  Nurses reported potential barriers to implementing pain management. Parent-related barriers include; attitudes, feasibility and competence.
  *C10: I think it would be a hard, um, thing to sell (topical anesthetics) to parents. To give any sort of pain medication that early in life. I mean a lot of patients don’t even want to take pain medications themself. Because they are afraid of the transfer through breast milk.*
  *C16: Well the only thing (is the sugar water)… I don’t know when they say one pack of sugar with 2 teaspoons of water. I don’t know in terms of the parents giving the baby, how convenient or how they’ll give it. Because people that use the syringe, sometimes, I am afraid that their baby will aspirate.*
  Some nurses reported barriers due to physical limitations of mothers (e.g., fatigue, pain) or ‘information barrage.’
  *C14: …as long as the parents can help you out, they’re physically able to do that then…like the parents are really sick or they can’t get out of bed, they can’t really put a finger in the baby’s mouth, can’t breastfeed then its hard to ask them to help you to distract the baby or hold the baby.*
  *C21: you know, as it is these parents are so anxious and now you’re gonna tell them to prepare all this stuff. Like, more headache.*
  Clinician-related barriers included; attitudes, time, workload, scope of practice, and institutional support.
  *C22: …not every practitioner realistically will advocate, some are in a rush, some just don’t feel pain management is even important to a newborn - it’s still a big problem.*
  *C17: We aren’t doing that many vaccines anyway so I think most of its relevant but yeah some of it I think it’s for older babies. So, it doesn’t apply.*
  *C08: If this is important for mothers and patients, it should be important to every health care worker and the institution as well, to increase patient satisfaction. So we do need education and it should be encouraged that everybody should know methods of relieving pain.*
  Empowering parents was suggested as a way to improve pain management practices.
  *C04: Have something noticeable for the parents by their bedside, for them to see that this is what your nurse should be doing… A nice colourful poster… That way it empowers the parents and reminds the nurse that these are the tasks that need to be done.*

### Changes to organization and content of the pamphlet

**Figure 1:**
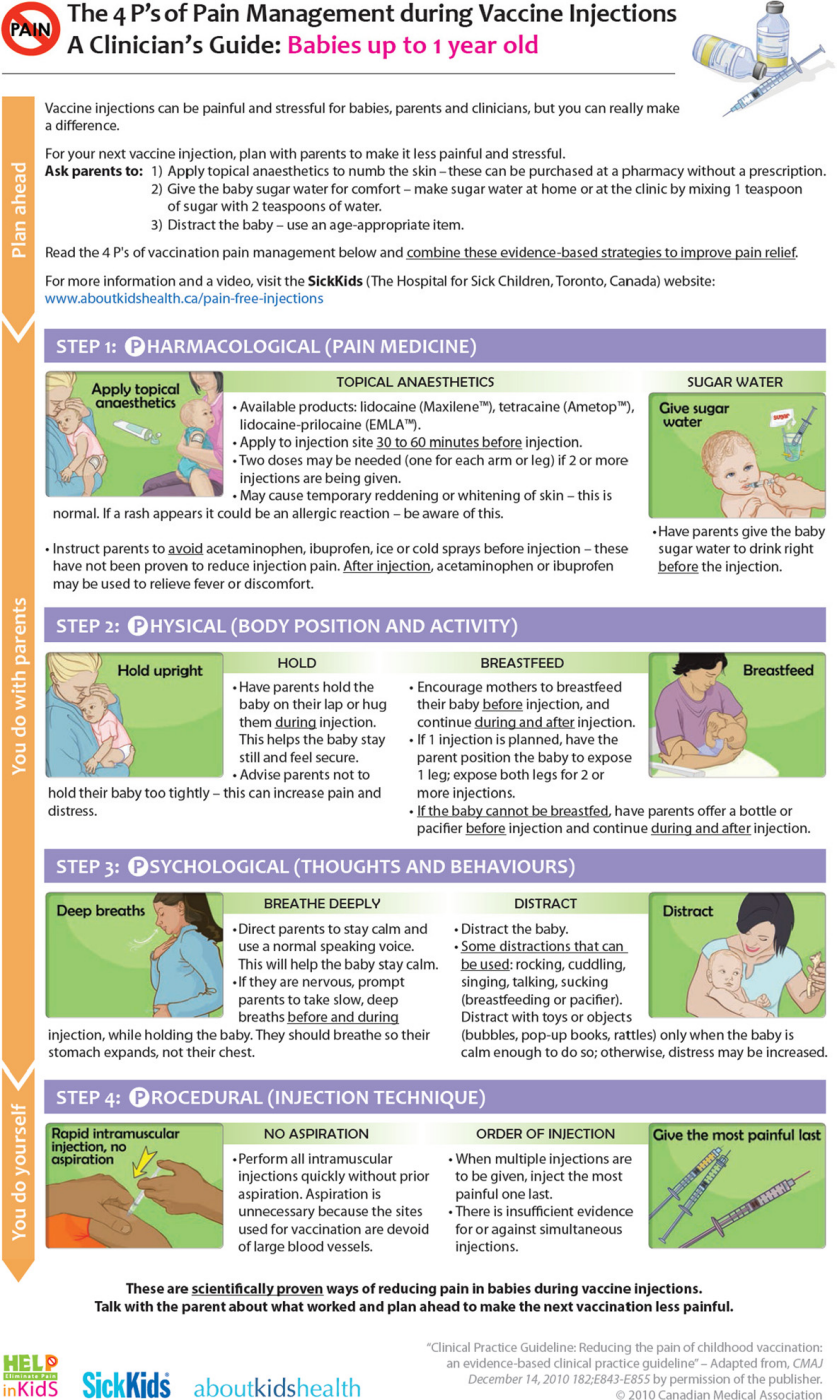
**Final version of pamphlet.**

After each round of interviews, iterative changes were made to the pamphlet based on feedback obtained from nurses. After the first round, the information for children > 12 months was removed. After the second round, the information was condensed to 1 page to reduce redundancy and the need to turn the page, and re-organized according to 4 types of pain management interventions (i.e., 4P’s: Pharmacological, Psychological, Physical and Procedural). In addition, interventions that clinicians carry out in collaboration with parents (i.e., pharmacological, psychological and physical) were specified versus those that clinicians are required to carry out on their own (procedural). A preparation section was placed at the top along with a website address where the information was posted to improve perceptions of the credibility of the information. Finally, names of commercially available topical anesthetic products were added to facilitate acquisition by parents. Additional edits were made after the third round of interviews to improve clarity and quality of images, reduce the amount of colour and harmonize the punctuation and writing style. The final version is shown in Figure 1.

### Feedback about the video

Suggestions for the video focused on reducing repetition and harmonizing the script and/or order of presentation with the pamphlet, to address perceptions that the video was too long and/or repetitive and that descriptions and/or order of presentation was inconsistent with the pamphlet. These suggestions were used to guide production of a revised video.

### Quantitative analysis – structured feedback and conceptual knowledge

The majority of nurses reported they understood all of the information in the pamphlet and video, and that the amount of information was ‘just right’ in terms of breadth and depth (Table 2). There was no difference for the different versions of the pamphlet (p > 0.05).

**Table 2:** **Structured feedback for pamphlet and video (n = 28)**

	**Understood all of the information in pamphlet (%)**	**Adequate information in pamphlet (%)**	**Understood all of the information in video (%)**	**Adequate information in video (%)**
Frequency, (%)	24 (85)	24 (85)	24 (85)	25 (89)

The knowledge test scores for the baseline, post-pamphlet, and post-video phases are shown in Table 3. The mean number of correct responses increased from baseline to post-pamphlet, and from post-pamphlet to post-video; p = 0.001 and p = 0.035, respectively. If only answers whereby nurses reported both the correct response and complete certainty in their level of confidence regarding their response were included, then the score similarly increased between the baseline and post-pamphlet phases, and between the post-pamphlet and post-video phases; p < 0.001 and p = 0.002, respectively. There was a non-significant effect of round of interview (1, 2, 3); that is, there was no difference for the different versions of the pamphlet; p = 0.37 and p = 0.25, respectively.

**Table 3:** **Nurses’ knowledge test scores (n = 28)**

	**Baseline**	**After pamphlet**	**After video**	P-value (baseline to pamphlet)**	P-value (pamphlet to video)**
Correct responses out of 12 questions*					
Correct	7.9 (2.0)	9.3 (2.3)	10.1 (1.2)	0.001	0.035
Correct & sure	3.9 (1.9)	7.5 (2.6)	8.6 (1.6)	<0.001	0.002
Correct responses out of 8 questions that correspond to information that was featured prominently in the educational material*					
Correct	6.1 (1.5)	6.9 (1.6)	7.6 (0.6)	0.011	0.029
Correct & sure	3.4 (1.7)	6.1 (2.1)	7.1 (1.2)	<0.001	0.001

*Values are mean and standard deviation (SD).

**Repeated measures ANOVA.

There was a similar pattern of results when only the subset of questions pertaining to information featured prominently in the education materials was included in the analysis (Table 3). The pattern of responses for individual questions are shown in Table 4.

**Table 4:** **Correct and sure responses for specific knowledge questions (n = 28)**

	**Frequency at baseline (%)**	**Frequency after review of pamphlet and video (%)**
1. Giving sugar water can reduce pain and distress.*	13 (46)	26 (93)
2. Using medicines like acetaminophen (Tylenol, Tempra), or ibuprofen (Advil, Motrin) can reduce pain and distress.	0 (0)	0 (0)
3. Putting ice on the skin can reduce pain and distress.	2 (7)	13 (46)
4. Breastfeeding can reduce pain and distress.*	21 (75)	27 (96)
5. Bottle feeding can reduce pain and distress.	6 (21)	17 (61)
6. Holding the baby can reduce pain and distress.*	18 (64)	27 (96)
7. Using numbing (anaesthetic) medicines can reduce pain and distress.*	15 (54)	25 (89)
8. Distracting the baby can reduce pain and distress.*	7 (25)	26 (93)
9. Acting calm can reduce pain and distress.*	8 (29)	24 (86)
10. Rubbing the skin can reduce pain and distress.	6 (21)	12 (43)
11. Performing intramuscular injections quickly without prior aspiration can reduce pain and distress.*	4 (14)	25 (89)
12. Giving the most painful vaccine first if multiple vaccines are injected sequentially can reduce pain and distress.*	9 (32)	20 (71)

*Information featured prominently in the educational material.

## Discussion

The current routine of under-treating vaccination pain in children is associated with significant child distress and parental dissatisfaction with the immunization experience [16]. Parents have expressed a desire to learn about reducing vaccination pain in their children and have identified the postnatal setting as an optimal environment for this education [9,12]. In order to address this care gap, we developed a CPG and template educational tools (pamphlet and video) for clinicians and parents [11]. According to the Knowledge-to-Action Framework [10], customization of tools to the local context is a critical aspect of translating research knowledge into improved practices. In this study, we adapted the pamphlet and video to meet the needs of postnatal nurses and evaluated usability and knowledge acquisition.

The results demonstrated nurses were satisfied with the educational tools and learned from them. They used institutional policies and practices as a reference point to judge the validity of the information. They identified a variety of dissemination methods to reach peers and new parents. For peers, they suggested e-modules, in-services, classroom video viewing, and poster displays in strategic locations. For parents, they suggested poster displays in patient rooms, video viewing in the lounge and direct dissemination of the pamphlets to parents. In addition, they expressed a preference for having access to the pamphlet and video, rather than the pamphlet alone, as they viewed them as complimentary. This feedback informed a clinical trial that is currently underway aiming at educating new parents on the postpartum ward about vaccination pain management in infants and evaluating the impact of this education on subsequent use of pain treatments during infant vaccinations.

Nurses reported wide variability in their practices with respect to the process of carrying out painful procedures in newborn infants, and inconsistent involvement of parents. Pain management was not explicitly discussed in interactions with parents. After review of the tools, nurses reported having a greater awareness of pain and pain management and opportunities to incorporate the information in their current practice. However, they identified barriers to implementation in parents and peers, including; attitudes, competence, and feasibility.

Significant improvement in nurses’ knowledge about evidence-based pain management strategies and in their confidence level in knowledge was also observed. In the baseline phase, nurses were largely uncertain about most of the evidence-based pain management options, scoring an average of 33% on the knowledge test when both correct responses and level of certainty of response were considered. This score increased significantly after review of the pamphlet (63%) and video (72%) by a relative amount of 90% and 120%, respectively. A similar improvement was observed when only questions pertaining to information that was prominently featured in the educational tools were included in the analysis. The results of this secondary analysis showed an average baseline score of 43% which subsequently rose to 76% after review of the pamphlet and then to 89% after review of the video. The relative improvement was 77% and 110%, respectively. We included nurse’s level of certainty in response in our knowledge test because nurses would not be expected to act on knowledge without being confident about it [12]. As this is the first study to evaluate knowledge uptake in clinicians from these materials, it serves as a benchmark for future work in this area. The results are consistent with the results observed in our companion study carried out in new parents hospitalized after the birth of an infant, whereby parents were satisfied with educational tools customized to their needs and preferences and demonstrated significant gains in knowledge after their review [12].

In prior research, we demonstrated that parents seek advice and endorsement by health care providers about vaccination pain management, including nurses [9,17]. We targeted postnatal nurses in a hospital setting because they routinely perform medical procedures in newborn infants, including vaccine injections, and educate new parents about baby health care topics. Opportunity therefore exists to incorporate education about vaccination pain management within current hospital education programs provided to new parents and takes advantage of information-seeking needs and motivation for learning. In addition, parents have specified the postnatal ward as a suitable setting to learn about infant vaccination pain management [12]. Under the guidance of postnatal nurses, parents can learn and practice the relevant skills (e.g., administering sugar water, breastfeeding) for implementing pain management during future infant vaccinations. Education of parents by nurses also reinforces education directed specifically to parents about this topic outside of the hospital setting and empowers parents to effect better pain management practices in their children during medical encounters involving painful procedures.

Two previous studies conducted in a public health setting and outpatient pediatric setting, respectively, demonstrated that educating clinicians about vaccination pain management led to greater utilization of analgesic interventions, higher levels of confidence in ability to mitigate pain, and greater satisfaction with pain management achieved during procedures [18,19]. To our knowledge, the impact of educating postnatal nurses on future parental pain management practices has not been previously explored and is worthy of study.

Some knowledge test questions were frequently answered incorrectly by nurses, even following review of the pamphlet and video. These questions related to the effectiveness of interventions not prominently displayed in the educational materials and/or that may have conflicted with nurses’ practices. It was also noted that some of the information contained in the tools was not consistent with institutional policies and practices (e.g., injecting intramuscular injections without aspiration). There is a need to harmonize information among the relevant sources accessed by nurses.

It is important to note that only a single hospital was included and only nurses working on the day shift on days when the study was being carried out could participate. It is possible that not all nurses’ perspectives were identified. In addition, the implementability of the educational tools in different postnatal settings is not known. Future studies are recommended to evaluate the feasibility and impact of implementing these tools within the postnatal setting.

Strengths of the study include the high recruitment rate and methodologic rigor. First, the recruitment rate was >90% of eligible participants approached for participation; this reduces the risk that the results are not generalizable to the wider population of postnatal hospital nurses. Second, the study design included an in-depth exploration of the usability of the educational tools and objective measures of the effectiveness of the tools. The qualitative component included a robust usability testing process, including a heuristic evaluation and three rounds of interviews to validate and improve the material. The involvement of the end-users (i.e., postnatal nurses) in this process improves the likelihood that the tools will be used [10]. The quantitative component included evaluation of knowledge about effective pain-relieving interventions. Demonstrating knowledge acquisition is a prerequisite to changes in behavior [10].

## Conclusion

In conclusion, the educational pamphlet and video about infant vaccine injection pain management was accepted by postnatal nurses and improved their knowledge about evidence-based pain-relieving interventions. Postnatal nurses can use the tools to improve their pain management practices and to educate parents about pain management for future infant vaccinations. Information gained from this study was used to inform the production of revised pamphlets and videos for children of different ages that are currently available on the Immunize Canada website at: http://immunize.ca/en/health-care-providers/painmgt.aspx.
